# The Induction of Heme Oxygenase 1 Decreases Painful Diabetic Neuropathy and Enhances the Antinociceptive Effects of Morphine in Diabetic Mice

**DOI:** 10.1371/journal.pone.0146427

**Published:** 2016-01-05

**Authors:** Sílvia Castany, Mireia Carcolé, Sergi Leánez, Olga Pol

**Affiliations:** Grup de Neurofarmacologia Molecular, Institut d’Investigació Biomèdica Sant Pau & Institut de Neurociències, Universitat Autònoma de Barcelona, Barcelona, Spain; The University of Hong Kong, HONG KONG

## Abstract

Painful diabetic neuropathy is a common complication of diabetes mellitus which is poorly controlled by conventional analgesics. This study investigates if treatment with an heme oxygenase 1 (HO-1) inducer, cobalt protoporphyrin IX (CoPP), could modulate the allodynia and hyperalgesia induced by diabetes and enhanced the antinociceptive effects of morphine. In a diabetic mice model induced by the injection of streptozotocin (STZ), we evaluated the antiallodynic and antihyperalgesic effects produced by the intraperitoneal administration of 5 and 10 mg/kg of CoPP at several days after its administration. The antinociceptive actions produced by the systemic administration of morphine alone or combined with CoPP were also evaluated. In addition, the effects of CoPP treatment on the expression of HO-1, the microglial activation marker (CD11b/c), the inducible nitric oxide synthase (NOS2) and μ-opioid receptors (MOR), were also assessed. Our results showed that the administration of 10 mg/kg of CoPP during 5 consecutive days completely blocked the mechanical and thermal hypersensitivity induced by diabetes. These effects are accompanied by the increased spinal cord, dorsal root ganglia and sciatic nerve protein levels of HO-1. In addition, the STZ-induced activation of microglia and overexpression of NOS2 in the spinal cord were inhibited by CoPP treatment. Furthermore, the antinociceptive effects of morphine were enhanced by CoPP treatment and reversed by the administration of an HO-1 inhibitor, tin protoporphyrin IX (SnPP). The spinal cord expression of MOR was also increased by CoPP treatment in diabetic mice. In conclusion, our data provide the first evidence that the induction of HO-1 attenuated STZ-induced painful diabetic neuropathy and enhanced the antinociceptive effects of morphine via inhibition of microglia activation and NOS2 overexpression as well as by increasing the spinal cord levels of MOR. This study proposes the administration of CoPP alone or combined with morphine as an interesting therapeutic approach for the treatment of painful diabetic neuropathy.

## Introduction

Neuropathic pain is one of the most common complications of diabetes, occurring in nearly 50% of patients [1] and remains an important clinical problem due to resistance to classical opioid analgesic drugs, such as morphine [2–6]. This loss in antinociceptive efficacy was described in diabetic animals following the systemic [2, 7], spinal [5, 8] and supraspinal [8] administration of μ-opioid receptor (MOR) agonists. As a consequence, high doses of morphine are required to inhibit neuropathic pain resulting in the induction of several side effects such sedation, respiratory depression, constipation and tolerance, among others [9].

Different mechanisms involving neuronal and non-neuronal factors were postulated to be responsible for the reduction in MOR agonists antinociceptive efficacy during painful diabetic neuropathy [4, 5, 10, 11]. That is, while several studies have demonstrated that this effect was associated with a loss of MOR and/or an impaired G-protein coupling to MOR [5, 10]. Other studies suggested that the activation of non-neuronal cells in the spinal cord, such as microglia, participates in the reduced antinociceptive effects produced by morphine under diabetic pain conditions. Indeed, the administration of minocycline, an specific inhibitor of microglia, potentiated the analgesic activity of morphine in STZ-induced diabetic neuropathy in mice [11]. Nevertheless, the search of new strategies to treat painful diabetic neuropathy and/or improve the analgesic effects of morphine during diabetic neuropathy is required.

Carbon monoxide is a gaseous neurotransmitter synthesized by the inducible (HO-1) and constitutive heme oxygenase enzymes. Several studies have shown that HO-1 over-expression or induction is associated with potent anti-inflammatory and antinociceptive effects [12–15]. Indeed, the administration of HO-1 inducer compounds, such as cobalt protoporphyrin IX (CoPP) inhibits acute and chronic pain. That is, the systemic administration of CoPP inhibits acute thermal nociception [16], inflammatory pain induced by the peripheral injection of formalin [17, 18] and the acetic acid-induced visceral pain [18]. In chronic pain, the administration of CoPP also inhibited the mechanical and thermal hypersensitivity induced by knee and paw inflammation [18, 19] among to those induced by the partial or total sciatic nerve injury as well as by the vincristine-induced neuropathic pain [14, 18, 20]. However the possible antinociceptive effects produced by CoPP treatment during painful diabetic neuropathy has not been evaluated.

We have also recently demonstrated that the administration of CoPP significantly enhanced the antiallodynic and antihyperalgesic effects produced by the local administration of morphine in acute thermal [16] and chronic inflammatory [18, 19] or nerve injury-induced neuropathic pain [18, 21]. Moreover, its antinociceptive effects were significantly decreased by the administration of the HO-1 inhibitor tin protoporphyrin IX (SnPP), indicating that HO-1 participates in the analgesic effects produced by morphine during acute and chronic inflammatory or nerve-injury induced neuropathic pain. Nevertheless, the role played by CoPP treatment on the antinociceptive effects of morphine during painful diabetic neuropathy and the possible mechanism involved in this action have not been evaluated.

In the other hand, it is well accepted that the spinal cord microglial cells are a key player in the etiology of diabetic neuropathic pain [22, 23]. That is hyperglycemia activated microglia and intracellular signaling molecules that are implicated in microglial functions including intracellular kinases (e.g., mitogen-activated protein kinases, MAPKs), and the release a variety of inflammatory mediators (such as proinflammatory cytokines, chemokines, nitric oxide, reactive oxygen species, etc.), which amplify the nociceptive signals in the spinal cord [24–27]. Oxidative stress and changes in nitric oxide formation played major roles in the onset of diabetic complications. Nitric oxide produced by the inducible nitric oxide synthase (NOS2) has been implicated in diabetic neuropathy as demonstrated by the reduced allodynia observed in diabetic NOS2 deficient mice [28]. In addition, previous studies have demonstrated that the exogenous induction of HO-1 alleviated neuropathic pain induced by sciatic nerve injury by reducing spinal cord microglia activation and nitric oxide synthesis [14]. However, whether HO-1 induction might avoid spinal microglial activation and reduces the liberation of inflammatory mediators, such us nitric oxide synthetized by NOS2 in diabetic neuropathy, remained to be investigated.

In the present study, using a mouse model of painful diabetic neuropathy induced by the intraperitoneal administration of streptozotocin (STZ), we evaluated: 1) the antiallodynic and antihyperalgesic effects produced by the intraperitoneal administration of CoPP; 2) the antinociceptive effects of the subcutaneous administration of morphine (a specific MOR agonist) alone and combined with CoPP; 2) the reversion of the antinociceptive effects of morphine with the HO-1 inhibitor, SnPP and 3) the effects of CoPP on the protein levels of HO-1 in the spinal cord, dorsal root ganglia and sciatic nerve as well as of those of CD11b/c (as a marker of microglial activation), NOS2 and MOR in the spinal cord from STZ-injected mice.

## Materials and Methods

### Animals

In vivo experiments were performed in male C57BL/6J mice acquired from Harlan Laboratories (Barcelona, Spain). All mice between 8 to 10 weeks old and weighing 21 to 25 g were housed under 12-h/12-h light/ dark conditions in a room with controlled temperature (22°C) and humidity (66%). Animals had free access to food and water and were used after a minimum of 6 days acclimatization to the housing conditions. All experiments were conducted between 9:00 AM and 5:00 PM. All experimental procedures within this study were carried out in accordance with the recommendations in the Guide for the Care and Use of Laboratory Animals of the National Institutes of Health. The protocol was approved by the local Ethical Committee of our Institution (Comissió d’Etica en l’Experimentació Animal i Humana de la Universitat Autònoma de Barcelona). This study was carried out in strict accordance with Universitat Autònoma de Barcelona (Permit Number: 6266). All efforts were made to minimize animal suffering and to reduce the number of animals used.

### Induction of Painful Diabetic Neuropathy

Diabetes was induced by the intraperitoneal administration of five consecutive daily injections of 55 mg/kg of streptozotocin (STZ; Sigma-Aldrich, St. Louis, MO) freshly prepared in citrate buffer (0.1M, pH 4.5) [29]. Control animals received an equal volume of citrate buffer alone (CTRL). Animals were fasted prior to the first administration of STZ and were allowed to feed again after injection. At 21 days after the first injection of STZ, the tail vein blood glucose levels were measured to confirm hyperglycemia by using a glucometer (OneTouch^®^ UltraMini^®^). In accordance to the recommendations established by [30], in this study we avoided the use of tests that animals restraint was required during measurements and employed multiple behavioral tests to identify the development of painful diabetic neuropathy in mice. That is, the development of mechanical allodynia, thermal hyperalgesia and thermal allodynia was evaluated by using the von Frey filaments, plantar and cold plate tests, respectively.

### Nociceptive Behavioral Tests

Mechanical allodynia was quantified by measuring the hind paw withdrawal response to von Frey filament stimulation. In brief, animals were placed in methacrylate cylinders (20 cm high, 9 cm diameter; Servei Estació, Barcelona, Spain) with a wire grid bottom through which the von Frey filaments (North Coast Medical, Inc., San Jose, CA) with a bending force in the range of 0.008–3.5 g were applied by using a modified version of the up–down paradigm, as previously reported by Chaplan et al. (1994) [31]. The filament of 3.0 g was used as a cut-off. Then, the strength of the next filament was decreased or increased according to the response. The threshold of response was calculated from the sequence of filament strength used during the up–down procedure by using an Excel program (Microsoft Iberia SRL, Barcelona, Spain) that includes curve fitting of the data. Clear paw withdrawal, shaking, or licking of the paw was considered as a nociceptive-like response. Both ipsilateral and contralateral hind paws were tested. Animals were allowed to habituate for 1 h before testing in order to allow an appropriate behavioral immobility.

Thermal hyperalgesia was assessed as previously reported by Hargreaves et al. (1988) [32]. Paw withdrawal latency in response to radiant heat was measured using the plantar test apparatus (Ugo Basile, Varese, Italy). Briefly, mice were placed in methacrylate cylinders (20 cm high x 9 cm diameter) positioned on a glass surface. The heat source was positioned under the plantar surface of the hind paw and activated with a light beam intensity. A cut-off time of 12 s was used to prevent tissue damage in absence of response. The mean paw withdrawal latencies from the ipsilateral and contralateral hind paws were determined from the average of three separate trials, taken at 5 min intervals to prevent thermal sensitization and behavioral disturbances. Animals were habituated to the environment for 1 h before the experiment to become quiet and to allow testing.

Thermal allodynia to cold stimulus was assessed by using the hot/cold-plate analgesia meter (Ugo Basile), previously described by Bennett and Xie (1988) [33]. The number of elevations of each hind paw was recorded in the mice exposed to the cold plate (4 ± 0.5°C) for 5 min.

### Western Blot Analysis

CTRL animals and STZ-induced diabetes mice treated with vehicle or CoPP were killed by cervical dislocation and tissues from the lumbar section of the spinal cord, dorsal root ganglia and sciatic nerves were removed immediately after sacrifice, frozen in liquid nitrogen, and stored at -80°C until assay. Samples from the spinal cord, dorsal root ganglia and sciatic nerves of two and/or three animals were pooled into one experimental sample to obtain enough protein levels for performing the Western blot analysis. The HO-1, CD11b/c, NOS2 and MOR protein levels were analyzed by Western blot.

For the evaluation of HO-1, CD11b/c, NOS2, total proteins were extracted. Then tissues were homogenized in ice-cold lysis buffer (50 mM Tris Base, 150 nM NaCl, 1% NP-40, 2 mM EDTA, 1 mM phenylmethylsulfonyl fluoride, 0.5 Triton X-100, 0.1% sodium dodecyl sulfate, 1 mM Na_3_VO_4_, 25 mM NaF, 0.5% protease inhibitor cocktail, and 1% phosphatase inhibitor cocktail). All reagents were purchased at Sigma (St. Louis, MO) with the exception of NP-40 from Calbiochem (Darmstadt, Germany). The crude homogenate was solubilised for 1 h at 4°C, sonicated for 10 s and centrifugated at 4°C for 15 min at 700 g. The supernatant was stored a -80°C.

For MOR, membrane proteins were extracted. Then, tissues were homogenized in ice-cold assay buffer (50 mM Tris-HCl, 1 mM EGTA and 5 mM MgCl_2_, pH 7.4) and centrifuged twice at 48.000 g at 4°C for 20 minutes. The pellet was resuspended in assay buffer and stored a -80°C.

Therefore, 60 μg of total or membrane proteins were mixed with 4 x laemmli loading buffer and then loaded onto 4% stacking/10% separating sodium dodecyl sulfate polyacrylamide gels.

The proteins were electrophoretically transferred onto PVDF membrane for 120 min, blocked with PBST + 5% nonfat dry milk, and subsequently incubated overnight at 4°C with polyclonal rabbit anti-HO-1 (1:300, Stressgen, Ann Arbor, MI); anti-CD11b/c (1:300, Novus Biologicals, Littleton, CO) antibody against the type 3 complement receptor to detect activated microglial cells; anti-NOS2 (1:200, Chemicon-Millipore) or anti-MOR (1:1000, Chemicon-Millipore, Billerica, MA) antibodies. The proteins were detected by a horseradish peroxidase-conjugated anti-rabbit secondary antibody (GE Healthcare, Little Chalfont, Buckinghamshire, United Kingdom) and visualized with chemiluminescence reagents (ECL kit; GE Healthcare) and by exposure onto hyperfilm (GE Healthcare). The intensity of blots was quantified by densitometry. The membranes were stripped and reproved with a monoclonal rabbit anti-β-actin antibody (1:10.000, Sigma) used as a loading control.

### Experimental design

In a first set of experiments, we assessed the expression of neuropathic pain induced by the intraperitoneal injection of STZ. After the habituation period, baseline responses were established in the following sequence: von Frey filaments, plantar and cold plate tests. After baseline measurements, diabetes was induced and animals were again tested in each paradigm at days 21 and 25 after STZ injection by using the same sequence as for baseline responses (n = 8 animals). Mice treated with an equal volume of citrate buffer (CTRL) were used as controls (n = 8 animals). Diabetic animals were randomly assigned to the treatment groups. Researchers who performed the behavioral tests were blinded regarding treatment received by the animals.

In a second set of experiments, we evaluated the mechanical antiallodynic, thermal antihyperalgesic and thermal antiallodynic effects of the intraperitoneal administration of 5 and 10 mg/kg of CoPP or vehicle in diabetic mice at 21 and 25 days after STZ-injection (n = 6 animals per group). In a third set of experiments the mechanical antiallodynic, thermal antihyperalgesic and thermal antiallodynic effects produced by the subcutaneous administration of different doses of morphine (0.5–10 mg/kg) or saline in diabetic mice at 21 days after STZ injection animals were also assessed (n = 6 animals per group). In a four set of experiments, we investigated the antinociceptive effects produced by the intraperitoneal administration of 10 mg/kg of CoPP combined with the subcutaneous administration of a low dose of morphine (0.5 mg/kg) in STZ-injected animals (n = 6 animals per group) as well as the reversion of the antiallodynic and antihyperalgesic effects produced by a high dose of morphine (10 mg/kg) by the administration of 10 mg/kg of SnPP intraperitoneally administered (n = 6 animals per group). All of these experiments were performed at 21 days after STZ-injection.

The doses of CoPP and SnPP were selected in accordance to our previous pilot studies as well as to other works [19, 21]. The doses of morphine administered were chosen from the dose-response curves performed in this study, as the ones that produced a minimal or a maximal antinociceptive effect in STZ-injected mice.

Finally, in another set of experiments we evaluated the effects of CoPP treatment on the expression of HO-1 in the lumbar section of the spinal cord, dorsal root ganglia and sciatic nerves as well as of those of CD11b/c and NOS2 in the lumbar section of the spinal cord from STZ-injected mice by using Western blot assay. Tacking account that functional MOR are located in plasmatic membranes, the expression of MOR were evaluated in membrane preparations from the spinal cord tissues. In these experiments mice treated with citrate buffer (CTRL) have been used as controls (n = 4 samples per group).

### Drugs

Streptozotocin were purchased from Sigma. CoPP and SnPP were acquired from Frontier scientific (Livchem GmbH & Co, Frankfurt, Germany). CoPP and SnPP were dissolved in DMSO (1% solution in saline) and morphine in saline solution (0.9% NaCl). All drugs were freshly prepared before use and administered in a final volume of 10 ml/kg. CoPP and SnPP were intraperitoneally administered at 3–4 h (CoPP) and 30 min (SnPP) before behavioural testing. Morphine was administered subcutaneously 30 min before behavioral testing. For each group treated with a drug the respective control group received the same volume of vehicle.

### Statistical Analysis

Data are expressed as mean ± SEM. The statistical analysis was performed by using the SPSS (version 17 for Windows, IBM España, Madrid, Spain). All comparisons were run as two-tailed testing. The comparison of the glucose levels in STZ-injected mice *versus* citrate buffer treated mice was evaluated by using an unpaired Student t test. The comparison of the mechanical allodynia, thermal hyperalgesia, and thermal allodynia induced by administration of STZ plus vehicle or CoPP at different doses and time points was evaluated by using a two way ANOVA repeated measures followed by the corresponding one way ANOVA and the Student Newman Keuls test. The effects produced by the administration of several doses of morphine or saline were evaluated by using a one way ANOVA followed by the Student Newman Keuls test. For each behavioral test, the comparison of the effects produced by the administration of CoPP or SnPP on the antinociceptive actions of morphine was also evaluated by using a one way ANOVA followed by the Student Newman Keuls test.

In these experiments, mechanical allodynia is expressed as the von Frey filaments strength (g), thermal hyperalgesia as the withdrawal latency (s) and thermal allodynia as the number of hind paws lifts.

Changes on the expression of HO-1, CD11b/c, NOS2 and MOR from CTRL and STZ-injected mice treated with vehicle or CoPP were analyzed by using a one way ANOVA followed by the Student Newman Keuls test. A value of p<0.05 was considered as a significant.

## Results

### Induction of diabetic neuropathy

In accordance to other reports, the administration of STZ produced weight loss (26.1 ± 0.6 g in CTRL mice *versus* 23.6 ± 0.5 g in STZ-injected animals; p< 0.01; unpaired Student t test; n = 8 animals per group) and a significant increase in plasma glucose levels (23.2 ± 0.4 mmol/l in STZ-injected animals *versus* 7.9 ± 0.2 mmol/l in CTRL mice; p<0.001; unpaired Student t test; n = 8 animals per group).

### Effect of CoPP treatment on the development of diabetic neuropathy

**Fig 1:**
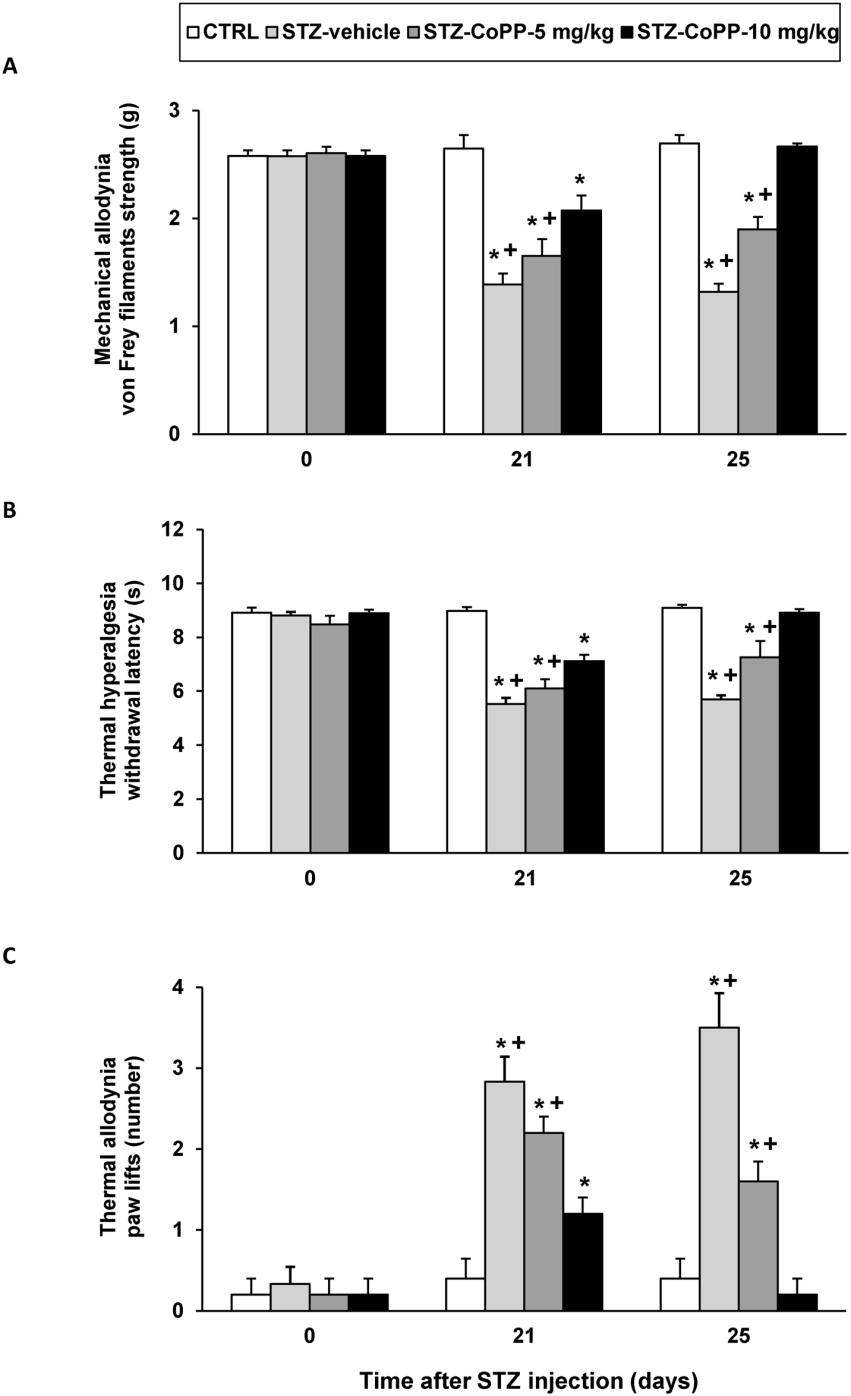
The antinociceptive effects produced by the intraperitoneal administration of CoPP at 5 and 10 mg/kg in STZ injected mice. The development of mechanical allodynia (A), thermal hyperalgesia (B) and thermal allodynia (C) in the hind paws of control and diabetic mice intraperitoneally treated with vehicle or CoPP at 5 and 10 mg/kg from day 21 to day 25 after STZ injection is shown. Data of tests are shown at day 0 (before diabetes induction) and at days 21 and 25 after STZ injection (one and five days after initiation of CoPP administration, respectively). Data are expressed as von Frey filaments strength (g) for mechanical allodynia, withdrawal latency (s) for thermal hyperalgesia and paw lifts (number) for thermal allodynia. For each day, * indicates significant differences vs. CTRL mice (p< 0.05, one-way ANOVA followed by the Student Newman Keuls test) and + indicates significant differences vs. STZ mice treated with CoPP at 10 mg/kg (p< 0.05, one-way ANOVA followed by the Student Newman Keuls test). The results are shown as the mean values ± SEM; n = 6–8 animals per group.

Animals were intraperitoneally daily treated with CoPP at 5 and 10 mg/kg or vehicle for a period of 5 days. On days 1 and 5 of treatment, the mechanical allodynia, thermal hyperalgesia and thermal allodynia were assessed. For all tests the two-way ANOVA repeated measures revealed a significant effect of the treatment (p<0.001) and time (p<0.001), and a significant interaction between them (p<0.001). That is, the significant decrease of the threshold for evoking hind paw withdrawal to a mechanical stimulus (Fig 1, panel A) and to a thermal stimulus (Fig 1, panel B) as well as the increased number of paw lifts during cold thermal stimulation (Fig 1, panel C) observed in STZ-vehicle treated mice were significantly attenuated or completely blocked in animals treated with CoPP at 5 and 10 mg/kg during 5 consecutive days, respectively. Moreover, while the mechanical and thermal allodynia as well as hyperalgesia induced by the administration of STZ were attenuated in animals treated with CoPP at 5 or 10 mg/kg during 1 day (p<0.001; one way ANOVA vs. CTRL animals), at 5 days of treatment, these nociceptive effects were only completely blocked in animals treated with 10 mg/kg of CoPP.

In CTRL mice, CoPP treatment did not produce any effect compared to mice treated with vehicle (data not shown).

Effects of the subcutaneous administration of morphine on the mechanical allodynia, thermal hyperalgesia and thermal allodynia induced by STZ injection in mice

**Fig 2:**
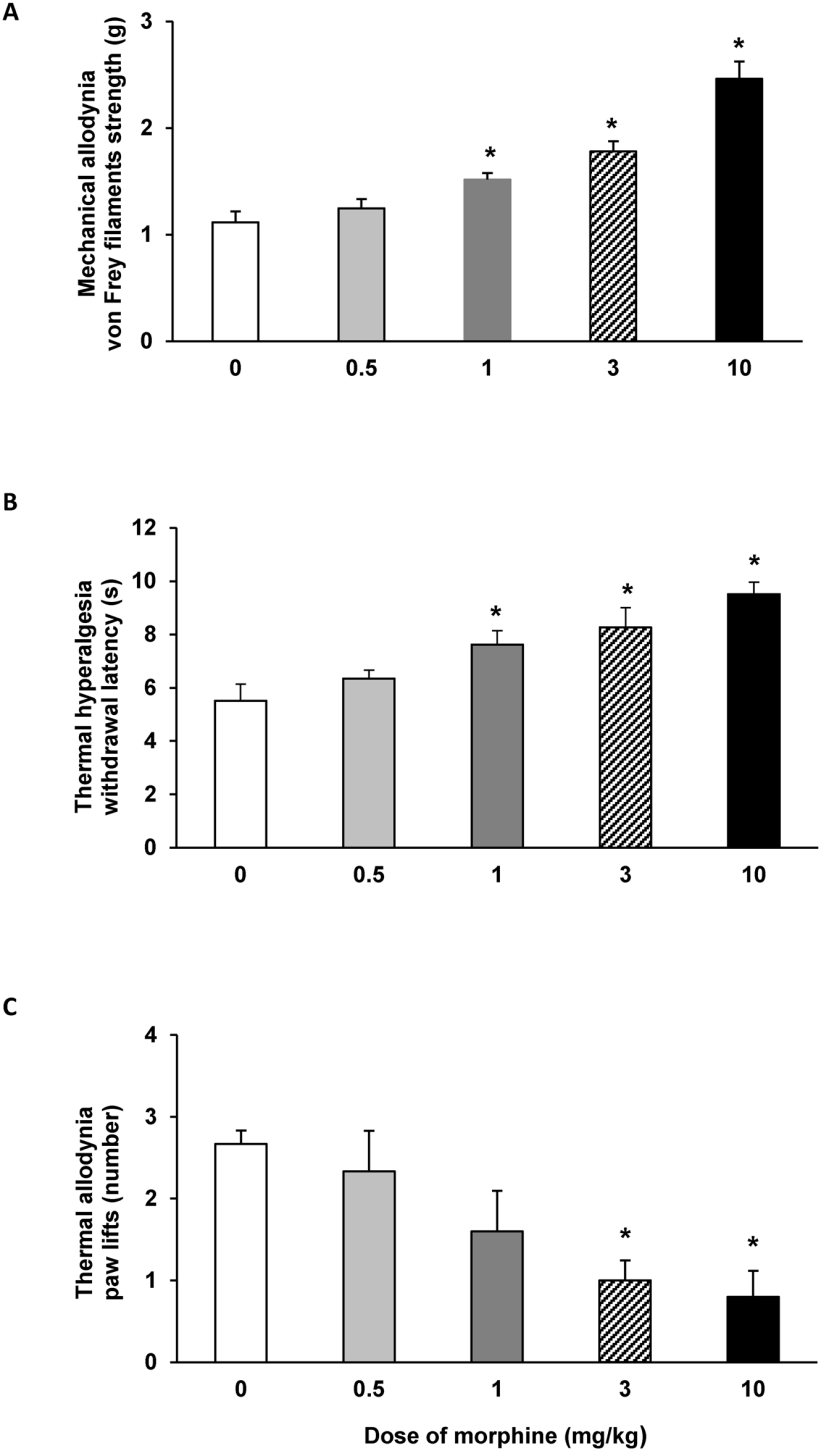
Effects of the subcutaneous administration of morphine on the mechanical allodynia, thermal hyperalgesia and thermal allodynia induced by the administration of STZ. Mechanical antiallodynic (A), thermal antihyperalgesic (B) and thermal antiallodynic (C) effects produced by the subcutaneous administration of different doses of morphine in STZ-injected mice. Data are expressed as von Frey filaments strength (g) for mechanical allodynia, withdrawal latency (s) for thermal hyperalgesia and paw lifts (number) for thermal allodynia. For each test, * denotes significant differences *versus* saline treated mice (0 mg/kg) (p< 0.05; one-way ANOVA followed by the Student Newman Keuls test). The results are shown as the mean values ± SEM; n = 6 animals for dose.

The subcutaneous administration of morphine (0.5–10 mg/kg) dose-dependently inhibited the mechanical allodynia (Fig 2A), thermal hyperalgesia (Fig 2B) and thermal allodynia (Fig 2C) induced by STZ injection. Indeed, the mechanical antiallodynic and thermal antihyperalgesic effects produced by the administration of morphine at 1, 3 and 10 mg/kg were higher than those produced by saline administration (p<0.001, one way ANOVA followed by the Student Newman Keuls test). In addition, the thermal antiallodynic effects produced by 3 and 10 mg/kg of morphine were also significantly different to those produced by saline (p<0.016, one way ANOVA followed by the Student Newman Keuls test).

### Effects of CoPP treatment on the antiallodynic and antihyperalgesic responses to morphine in STZ-injected mice

**Fig 3:**
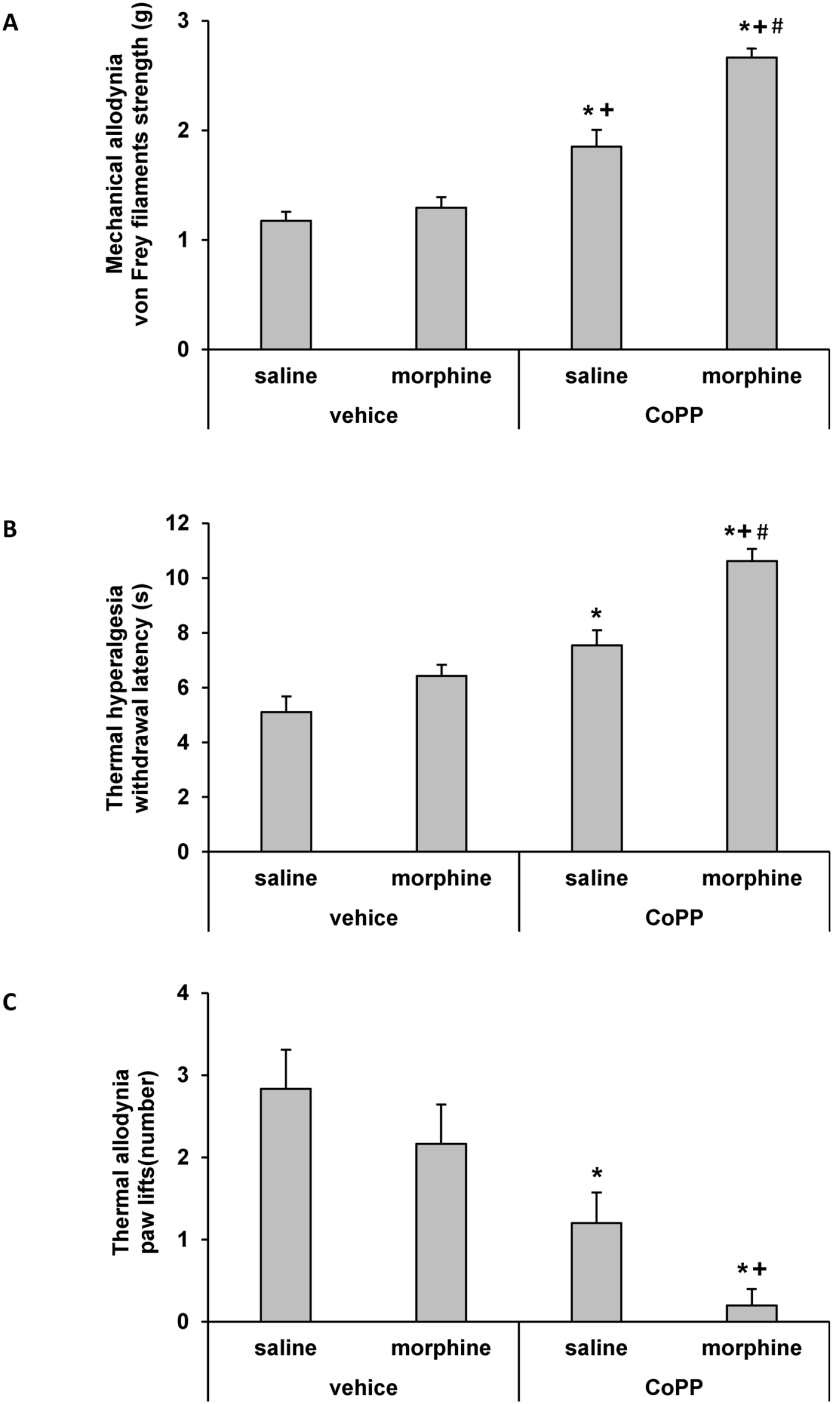
Effects of CoPP treatment on the antiallodynic and antihyperalgesic responses to morphine. Mechanical antiallodynic (A), thermal antihyperalgesic (B), and thermal antiallodynic (C) effects of the subcutaneous administration of 0.5 mg/kg of morphine or saline in STZ-injected mice pretreated with vehicle (DMSO 1%) or 10 mg/kg of CoPP. The effects of the intraperitoneal administration of CoPP alone are also shown. Data are expressed as von Frey filaments strength (g) for mechanical allodynia, withdrawal latency (s) for thermal hyperalgesia and paw lifts (number) for thermal allodynia. For each behavioral test, * denotes significant differences *versus* control group treated with vehicle plus saline (p< 0.05, one-way ANOVA followed by Student Newman Keuls test), + denotes significant differences *versus* group treated with vehicle plus morphine (p< 0.05, one-way ANOVA followed by the Student Newman Keuls test) and # denotes significant differences *versus* group treated with CoPP plus saline (p< 0.05; one-way ANOVA followed by the Student Newman Keuls test).

The effects of the intraperitoneal administration of 10 mg/kg of CoPP or vehicle (DMSO 1%) on the mechanical antiallodynic (Fig 3A), thermal antihyperalgesic (Fig 3B) and thermal antiallodynic (Fig 3C) effects produced by the subcutaneous administration of a low dose of morphine (0.5 mg/kg) or saline in STZ-injected mice were investigated. Our results showed that treatment with CoPP combined with the subcutaneous administration of a low dose of morphine significantly enhanced the mechanical antiallodynic (Fig 3A), thermal antihyperalgesic (Fig 3B) and thermal antiallodynic (Fig 3C) effects produced by morphine, CoPP or vehicle administered alone (p<0.001; one-way ANOVA followed by the Student Newman Keuls test).

### Reversal of the antinociceptive responses produced by morphine in STZ-injected mice with the administration of the HO-1 inhibitor, SnPP

The effects of the intraperitoneal administration of 10 mg/kg of SnPP or vehicle (DMSO 1%) on the inhibition of the mechanical allodynia, thermal hyperalgesia and thermal allodynia produced by the subcutaneous administration of a high dose of morphine (10 mg/kg) in STZ-injected mice are shown in Table 1. Our results showed that the coadministration of SnPP with a high dose of morphine significantly reversed the mechanical antiallodynic, thermal antihyperalgesic and thermal antiallodynic effects produced by this drug administered alone (p<0.001, one-way ANOVA followed by the Student Newman Keuls test). Moreover, the intraperitoneal administration of SnPP plus saline did not produce any mechanical antiallodynic, thermal antihyperalgesic and thermal antiallodynic effect as compared with vehicle plus saline treated mice.

**Table 1:** Mechanical response (von Frey filaments strength, g), thermal heat response (withdrawal latency, s) and thermal cold response (paw lifts, number) on the hind paws of STZ injected mice treated with vehicle-saline, vehicle-morphine, SnPP-saline or SnPP-morphine.

***Treatment***	***Mechanical response von Frey filaments strength (g)***	***Thermal heat response withdrawal latency (s)***	***Thermal cold response paw lifts (number)***
vehicle-saline	1.4 ± 0.1	5.1 ± 0.3	2.8 ± 0.2
vehicle-morphine	2.4 ± 0.1*	9.4 ± 0.3*	0.6 ± 0.4*
SnPP-saline	1.2 ± 0.1	5.8 ± 0.8	2.6 ± 0.9
SnPP-morphine	1.5 ± 0.1	5.7 ± 1.0	3.0 ± 0.7

Results are shown as mean values ± SEM; n = 6 animals per experimental group. For each test,

* p<0.001 denotes significant differences vs the other experimental groups (one way ANOVA followed the Student Newman Keuls test).

### Effect of CoPP treatment on HO-1, CD11b/c, NOS2 and MOR protein expression

**Fig 4:**
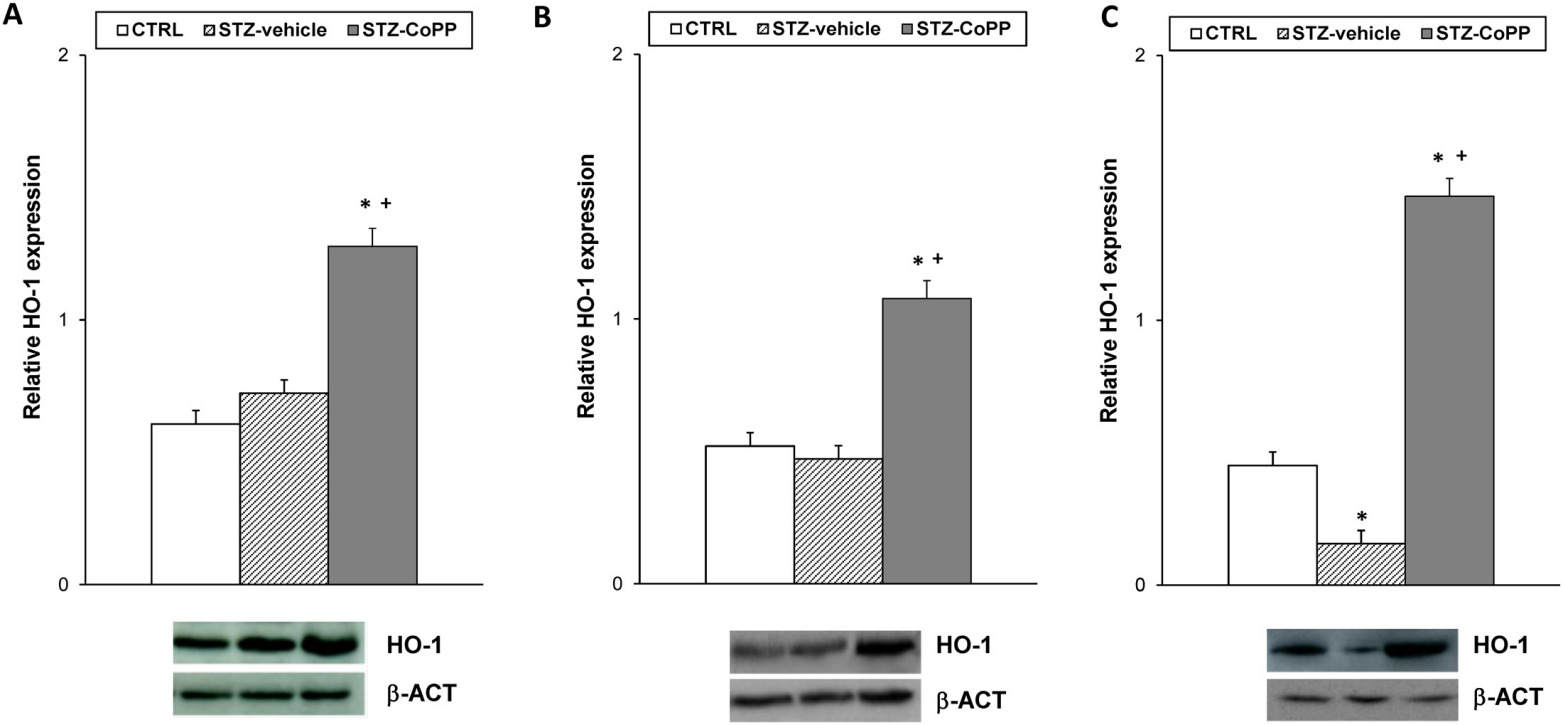
Effect of CoPP treatment on HO-1 protein expression in the spinal cord, dorsal root ganglia and sciatic nerve from STZ-injected mice. The protein expression of HO-1 in the spinal cord (A), dorsal root ganglia (B) and sciatic nerve (C) from STZ-injected mice treated with vehicle or CoPP is represented. The expression of HO-1 in the spinal cord, dorsal root ganglia and sciatic nerve from CTRL mice treated with vehicle has been also represented as controls. For each tissue, * indicates significant differences when compared to CTRL mice (p< 0.05, one-way ANOVA followed by Student Newman Keuls test) and + when compared to STZ vehicle treated mice (p< 0.05, one-way ANOVA followed by Student Newman Keuls test). Representative examples of western blots for HO-1 (32 kDa) in which β-actin (42 kDa) was used as a loading control, are also shown. Data are expressed as the relative expression ± SEM; n = 4 samples per group.

The protein levels of HO-1in the spinal cord, dorsal root ganglia and sciatic nerve from STZ-injected mice treated with vehicle or CoPP as well as from CTRL mice treated with vehicle are shown in Fig 4. Our results demonstrated that while the spinal cord (Fig 4A) and dorsal root ganglia (Fig 4B) protein levels of HO-1 were not altered in diabetic mice, a reduced expression was observed in sciatic nerves of diabetic mice as compared to CTRL animals (Fig 4C; p<0.001; one-way ANOVA followed by the Student Newman Keuls test). Nevertheless, a significant increase in HO-1 protein levels was observed in the spinal cord, dorsal root ganglia and sciatic nerves of diabetic mice treated with CoPP (p<0.025; one-way ANOVA, followed by the Student Newman Keuls test, *versus* CTRL and STZ vehicle treated mice).

**Fig 5:**
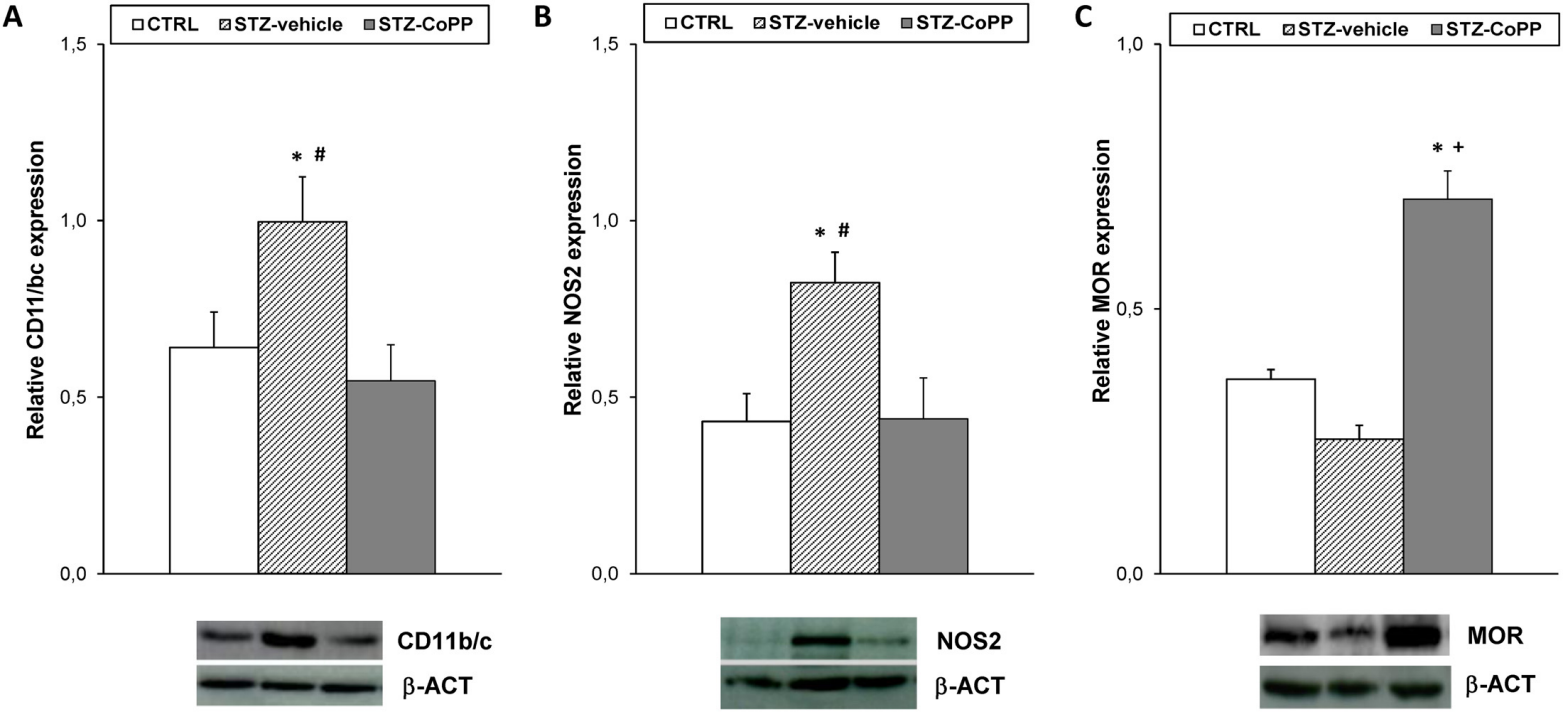
Effect of CoPP treatment on CD11b/c, NOS2 and MOR protein expression in the spinal cord from STZ-injected mice. The protein expression of CD11b/c (A), NOS2 (B) and MOR (C) in the spinal cord from STZ-injected mice treated with vehicle or CoPP is represented. The expression of CD11b/c, NOS2 and MOR from CTRL mice treated with vehicle has been also represented as controls. For each protein, * indicates significant differences when compared to CTRL animals (p< 0.05, one-way ANOVA followed by Student Newman Keuls test), + indicates significant differences when compared to STZ vehicle treated animals (p< 0.05, one-way ANOVA followed by Student Newman Keuls test) and # indicates significant differences when compared to STZ-CoPP treated animals (p< 0.05, one-way ANOVA followed by Student Newman Keuls test). Representative examples of western blots for CD11b/c (160 kDa), NOS2 (130 kDa) and MOR (50 kDa) proteins, in which β-actin (42 kDa) was used as a loading control, are also shown. Data are expressed as the relative expression ± SEM; n = 4 samples per group.

We also investigated whether the significant increased expression of CD11b/c and NOS2 in the spinal cord from STZ vehicle treated animals (p<0.010; one-way ANOVA *versus* CTRL vehicle treated mice) could be altered by CoPP treatment (Fig 5A and 5B). Our data revealed that CoPP treatment blocked the increased expression of CD11b/c and NOS2 induced by diabetes (p<0.017; one-way ANOVA *versus* STZ vehicle treated mice).

Our results also indicated that membrane MOR protein levels were marginally reduced in the spinal cord of diabetic mice and significantly increased in CoPP treated animals (p<0.001; one-way ANOVA *versus* CTRL and STZ vehicle treated mice; Fig 5C).

## Discussion

In this study, we demonstrated that the intraperitoneal administration of CoPP attenuated painful diabetic neuropathy and increased the antinociceptive effects of morphine. Furthermore, CoPP treatment enhanced the HO-1 and MOR levels and decreased the activation of microglia and the up-regulation of NOS2 in the spinal cord from diabetic mice.

Our results showed that the intraperitoneal injection of STZ in mice exhibited hyperglycemia, weight loss as well as to mechanical and thermal allodynia, and thermal hyperalgesia at 21 days after diabetes induction. Although this model is not probably the best to make its translation to human diabetic polyneuropathy due to the short duration of the experimental process [30] the presence of mechanical and thermal hypersensivity, as previously observed in other short-term diabetes models [34], supported its use for studying the antinociceptive actions produced by the administration of CoPP alone and combined with morphine during painful diabetic neuropathy.

CoPP is a well-established HO-1 inducer compound with recognized antinociceptive and antiinflammatory properties in several experimental models of inflammatory and neuropathic pain [14, 18–20, 35]. Our results showed for the first time that the repeated administration of CoPP completely abolished the mechanical and thermal allodynia as well as the thermal hyperalgesia induced by the injection of STZ. Consistently, the protein levels of HO-1 in the spinal cord, dorsal root ganglia and sciatic nerves from diabetic mice treated with CoPP were significantly increased. In accordance to these results, an increased expression of HO-1 in diabetic animals treated with several HO-1 inducer compounds have been also reported in different tissues such as, spinal cord, pancreas, kidney [36–38]. In all of these experiments an improvement of the principal symptoms of diabetes have been also revealed in CoPP treated animals, indicating that the increased expression of HO-1 induced by this treatment is the principal responsible for their neuroprotective effects in diabetic animals. We have previously demonstrated that the repeated intraperitoneal injection of CoPP also inhibited the sciatic nerve injury-induced neuropathic pain and inflammatory pain by increasing the expression of HO-1 [14, 19]. The present data indicate that HO-1 also plays an important role in mediating the antinociceptive effects of CoPP on neuropathic pain induced by diabetes mellitus.

Our results also showed that while the spinal cord and dorsal root ganglia protein levels of HO-1 were not altered by diabetes a significant reduction in its expression was observed in sciatic nerves of diabetic mice as compared to controls. These results are in contrast to the enhanced expression of HO-1 observed in the spinal cord of diabetic animals [36] but agree with the down-regulated protein levels of this enzyme shown in sciatic nerves of diabetic animals [39]. The possible discrepancies in the spinal cord levels of HO-1in diabetic mice could be probably related to the different evaluation times after diabetes induction (3 vs. 6 weeks) as well as the different molecular techniques used to evaluate their expression (western blot vs. immunohistochemistry). Nevertheless, the reduced HO-1 levels in sciatic nerve of diabetic animals is among the consequence of hyperglycemia that contribute to prevailing oxidative stress condition in peripheral nerves [39]. CoPP treatment enhanced the levels of this enzyme in sciatic nerves and spinal cord thus exerting a protective effect in diabetic neuropathy.

Several authors have demonstrated the important role played by spinal microglial cells in the development of diabetic neuropathy [22]. That is, activated microglia promotes the consolidation and progression of painful diabetic neuropathy by regulating the synthesis of several inflammatory mediators, such as nitric oxide. In this study we have also demonstrated that CoPP treatment inhibited microglial activation in the spinal cord from diabetic mice. Therefore and taking account the reduced behavioral symptoms of neuropathic pain observed in diabetic mice treated with selective inhibitors of microglial activation [11] and the reduced expression of CD11b/c (a marker of microglial activation) in the spinal cord of diabetic mice treated with CoPP, that we hypothesized that the antinociceptive effects of CoPP in diabetic mice might, at least in part, be mediated through the inhibition of the spinal microglial activation.

It is also well known that under neuropathic pain conditions, HO-1 may exert its antinociceptive effects via modulation the expression of NOS2 [14, 21]. Therefore, the reduced protein levels of NOS2 observed in the spinal cord from diabetic mice treated with CoPP suggests that the alleviation of diabetic neuropathy produced by this HO-1 inducer compound might be also due by blocking the spinal synthesis of nitric oxide produced by NOS2. We do not exclude the possibility that CoPP might also exert its anticonception effects via modulation of cGMP signaling pathway in spinal neurons [40] as well as by reducing the up-regulated expression of COX-2 as shown in other diabetic models [39, 41]. Nevertheless, because oxidative stress and inflammation play a pivotal role in the development and progression of diabetic complications, such us painful diabetic neuropathy [39], this study demonstrated that the activation of HO-1 may provide beneficial effects in neuropathy, as demonstrated by the inhibition of activated microglia and the synthesis of nitric oxide induced by diabetes.

In the present study, we also demonstrated for the first time that the antiallodynic and antihyperalgesic effects of subcutaneous administration of morphine in diabetic mice were significantly enhanced by CoPP treatment. This finding represents an interesting improvement in the therapy of painful diabetic neuropathy taking account the loss antinociceptive efficacy of morphine in these experimental conditions. Moreover, the effective antinociceptive effects produced by the combination of a small dose of morphine with CoPP might avoided the possible side effects produced by this opioid when was repetitively administered a high doses. In addition, the fact that the antinociceptive effects of morphine were completely reversed by the administration of an HO-1 inhibitor (SnPP) revealed the involvement of HO-1 in the inhibitory effects produced by morphine during painful diabetic neuropathy.

The possible mechanisms implicated in the increased antinociceptive effects of morphine produced by CoPP under painful diabetic neuropathy state are not elucidated. Our data demonstrated that while STZ-induced diabetes slightly reduced the membrane MOR levels in the spinal cord, and an enhanced expression of these receptors was observed in CoPP treated mice. That is, the expression of MOR in CoPP treated animals was higher than those obtained in controls and STZ-injected mice treated with vehicle, indicating that CoPP treatment not only avoided the diminished expression of MOR induced by diabetes but rather increased their protein in diabetic animals. Thus explaining the increased effectiveness of morphine in CoPP diabetic treated mice. These data are consistent with previous findings showing that the activation of HO-1 increased morphine effectiveness in animals with neuropathic pain induced by the chronic constriction of the sciatic nerve in mice by enhancing MOR expression [21]. Nevertheless, in addition to the enhanced expression of MOR produced by CoPP treatment, changes in the functional coupling of MOR to G proteins, in the density of distinct G protein subunits, etc. induced by the activation of HO-1 might not be excluded.

The non-significant changes in the protein levels of MOR in the spinal cord from diabetic mice treated with vehicle are in accordance to the unchanged expression of MOR observed in the majority of investigations performed after 3–4 weeks of STZ-induced diabetes [5, 42] but in contrast to the down-regulated expression of these receptors demonstrated by Shaqura et al., (2013) [43] in the spinal cord from diabetic rats at 12 weeks after STZ injection. These discrepancies are probably related to the different period of time allowed after STZ-induced diabetes, that is the early (3–4 weeks) and at more advanced stage (12 weeks).

It is also well known that treatment with selective inhibitors of microglial activation besides to reduce the behavioral symptoms of neuropathic pain in diabetic mice, also enhanced the antinociceptive effects of morphine systemically administered [11]. Therefore, the fact that CoPP treatment avoided the activation of microglia in diabetic mice indicated that the induction of HO-1 might also increase the antinociceptive effects of morphine via inhibiting microglia activation.

In conclusion, our results demonstrated that the induction of HO-1 might alleviate diabetic neuropathy and enhanced the antinociceptive effects of morphine via inhibition of microglia activation and NOS2 overexpression as well as by regulating MOR spinal cord expression. These data provide promising therapeutic approaches for the treatment of STZ-induced painful diabetic neuropathy.
